# Reply to C. Stewart’s Letter to the Editor Re: Teoh SL *et al.*, *Nutrients* 2016, 8, 57

**DOI:** 10.3390/nu8040228

**Published:** 2016-04-19

**Authors:** Siew Li Teoh, Suthinee Sudfangsai, Pisake Lumbiganon, Malinee Laopaiboon, Nai Ming Lai, Nathorn Chaiyakunapruk

**Affiliations:** 1 School of Pharmacy, Monash University Malaysia, Selangor 47500, Malaysia; charmaine.slteoh@gmail.com; 2 Faculty of Pharmaceutical Sciences, Naresuan University, Phitsanulok 65000, Thailand; suthinee_sfs@hotmail.com; 3 Department of Obstetrics and Gynaecology, Faculty of Medicine, Khon Kaen University, Khon Kaen 40002, Thailand; pisake@kku.ac.th; 4 Department of Biostatistics and Demography, Faculty of Public Health, Khon Kaen University, Khon Kaen 40002, Thailand; laopaiboonmalinee@yahoo.co.uk; 5 School of Medicine, Taylor’s University Malaysia, Selangor 47500, Malaysia; lainm123@yahoo.co.uk; 6 Center of Pharmaceutical Outcomes Research (CPOR), Department of Pharmacy Practice, Faculty of Pharmaceutical Sciences, Naresuan University, Phitsanulok 65000, Thailand; 7 School of Pharmacy, University of Wisconsin, Madison, WI 53705-2222, USA; 8 School of Population Health, University of Queensland, Brisbane, Herston 4006, Australia

In a recent systematic review and meta-analysis report (Nutrients 2016, 8, 57), we critically appraised and summarized current evidence to determine the effects of chicken essence in improving cognitive functions as well as its safety. Our report showed a lack of convincing evidence to support the cognitive-enhancing effects of chicken essence. A critique of our work appeared in *Nutrients* (Stewart C Letter to the Editor 2016, 8, 201). In this Reply, we have endeavored to respond to each point highlighted by Stewart, commencing with corrections to our original report.

## 1. Corrections

Stewart identified an error in our report—“Table 4 identifies Executive Function, WAIS arithmetic test, Azhar 2008 as significant, but the 95% CI includes zero”. We thank Stewart for identifying this error and wish to make the following corrections.

### 1.1. In Table 4, Executive Function Domain

The star denoting a significant difference in the WAIS-Arithmetic test in Azhar 2008 should be removed.

### 1.2. In Section 3.4. Effects of Chicken Essence in Cognitive Function Improvement—First Paragraph

“Of the 30 complete data, 12 (instead of 13) were found to have significant differences between CE group and placebo group, where seven (instead of eight) were in executive function domain, and five in short-term memory domain”.

## 2. Small Number of Studies Included

Stewart questions the exclusion of a large number of studies when the grounds for inclusion were very broad. The inclusion criteria were carefully defined using the PICO framework to be specific to our research question [1]. However, we acknowledge that the number of excluded studies at the screening stage is large. This is because we did not initially limit our search strategy to human randomized controlled trials (RCTs) and we searched using subject terms only [2].

## 3. Underpowered Clinical Trials and High Drop-Out Rates

Stewart asserts that including trials with small number of subjects and high drop-out rates provides unwarranted results. We agree that the number of subjects recruited in individual trials was relatively small. Therefore, the need to perform a meta-analysis is warranted as the power would be increased by combining multiple studies. We acknowledge that a high drop-out rate in a trial can cause attrition bias, which was fully assessed in “incomplete outcome reporting” domain of Cochrane’s risk of bias tool. Therefore, we would argue that we have presented the pooled findings with explicit consideration of its implication on our confidence in interpreting the findings instead of presenting unwarranted results.

## 4. Heterogeneity of Trials

### 4.1. Subject Characteristics and Chicken Essence Regimens

Stewart refutes combining cognitively impaired subjects with healthy subjects and combining trials with heterogeneous regimens of chicken essence (CE). In addition, he asserts that healthy subjects of young age were less likely to show a cognitive effect even if one existed. We acknowledged that the trials combined in our primary analysis had the heterogeneity issue. Therefore, we conducted further analysis to remove the heterogeneity by excluding the trial with subjects of poorer cognitive function and different CE regimen. The further analysis, which combined trials with healthy subjects of young age and similar CE regimens only, showed a pooled result with a significant difference and another pooled result with no significant difference [3].

### 4.2. Cognitive Function Tests and RCT Designs

Stewart refutes combining trials with different cognitive function tests. We acknowledge the differences in the cognitive function tests. Therefore, we pooled data only from the most comparable cognitive function tests and used standardized mean difference (SMD) to address the differences in measurement scales [4].

Stewart refutes combining trials with different designs. However, combining data from a parallel trial (The parallel trial in Table 5 of [3] should be Young 2015 (instead of Yamano 2015)) and a cross-over trial has been widely accepted [5].

## 5. Chicken Meat Ingredient-168 (CMI-168)

Stewart refutes the inclusion of the trial which used CMI-168 in the intervention group. However, CMI-168 was still considered as CE based on its description of “a hydrolyzed chicken extract prepared from chicken meat that had been processed by a proprietary technology” [6]. This definition was consistent with the definition of CE used in our report. Therefore, we remain assured that the trial was appropriate for inclusion.

## 6. Poor Quality of Trials

Stewart asserts that synthesizing evidence from poor quality trials provides unreliable results. We agree that the poor quality of trials reduced the confidence in the effect estimate and this was highlighted using the GRADE approach in our report. Therefore, we stressed in the report the necessity of interpreting the findings of the meta-analysis with caution.

## 7. Comparison with the Effect of Carnosine

Stewart refutes our decision to relate the effect of CE with the effect of carnosine. We agree that carnosine might not be exactly the same as CE. However, carnosine is documented as one of the primary active contents of CE [7,8,9]. We chose carnosine for comparison because it was the closest product to CE. The readers would benefit from seeing how our CE findings would be compared to a similar product.

## 8. Conclusions

In conclusion, we show in this reply that the issues raised by Stewart were not ignored but highlighted with sound methods. Our report is useful as an exploratory meta-analysis [10] to provide a plausible effect estimate and to identify gaps in the RCTs in evaluating the cognitive effects of CE.

We are grateful to Stewart for bringing our attention to some pertinent issues in the conduct of systematic review and meta-analysis, and for giving us an opportunity to reassess our work from his perspective.
